# Early Return to Play Following Conservative Treatment of a Lumbar Flexion-Distraction Injury in a Professional Soccer Player: A Case Report

**DOI:** 10.7759/cureus.84366

**Published:** 2025-05-18

**Authors:** Ryota Kimura, Yuichi Ono, Naohisa Miyakoshi

**Affiliations:** 1 Orthopedic Surgery, Akita University Graduate School of Medicine, Akita, JPN

**Keywords:** conservative treatment, posterior ligamentous complex injuries, professional athlete, soccer player, thoracolumbar fracture

## Abstract

Flexion-distraction injuries of the thoracolumbar spine, often classified as AO type B2, are typically considered unstable and are generally managed surgically. However, the optimal approach for managing such injuries in elite athletes remains unclear, particularly considering the potential career-limiting consequences of spinal fusion. We report the case of a 32-year-old professional soccer player who sustained a flexion-distraction injury at the T12-L1 level during a match. Imaging confirmed an L1 compression fracture with posterior ligament complex disruption. Despite surgical indications, the athlete exhibited excellent core stability, no neurological deficits, and preserved alignment. A multidisciplinary team opted for conservative treatment, including rigid bracing and staged rehabilitation. The athlete resumed non-contact training at week seven, returned to full-contact practice at week 10, and competed in official matches by week 12. At the two-year follow-up, he remained symptom-free, with no spinal deformity or recurrence. This case suggests that in elite athletes with preserved spinal stability and core strength, conservative management of flexion-distraction injuries may allow for a timely return to play while avoiding the long-term drawbacks of fusion surgery. Individualized decision-making and close follow-ups are essential.

## Introduction

Thoracolumbar spine trauma can significantly affect quality of life due to neurologic deficits, pain, and deformity. These injuries can result in long-term disability, loss of functional independence, and considerable socioeconomic impact. However, the epidemiology of lumbar spine trauma remains unknown. An expert panel convened by the World Federation of Neurosurgical Societies Spine Committee evaluated 39 studies on the epidemiology of thoracolumbar spine fractures, ultimately estimating an incidence of 30 per 100,000 individuals, including osteoporotic fractures [1].

Airborne, alpine winter, and water sports are high-risk activities for spinal trauma among sports-related causes [2-4]. Although sports-related spinal injuries are relatively uncommon, their potential to disrupt careers makes them particularly concerning for elite athletes.

Flexion-distraction injuries of the thoracolumbar spine, such as Chance fractures, often result from high-energy trauma and involve disruption of the posterior ligamentous complex. This three-column injury is typically managed surgically. The AO Spine Thoracolumbar Injury Classification System defines type B2 injuries as unstable and often requiring surgical fixation [5].

However, spinal fusion in elite athletes remains a significant clinical challenge. Although the procedure can provide stability and pain relief, it may also result in reduced spinal mobility, which can be particularly detrimental for athletes who rely on a full range of motion for optimal performance. Furthermore, the recovery process may be prolonged, potentially delaying return to play (RTP) and affecting the athlete’s career trajectory. In addition, there is a heightened risk of implant failure due to high physical demands, as well as the possibility of adjacent segment degeneration over time, which could necessitate further intervention. In high-demand sports such as soccer, spinal instrumentation can be detrimental to performance and potentially end careers [6].

## Case presentation

**Figure 1:**
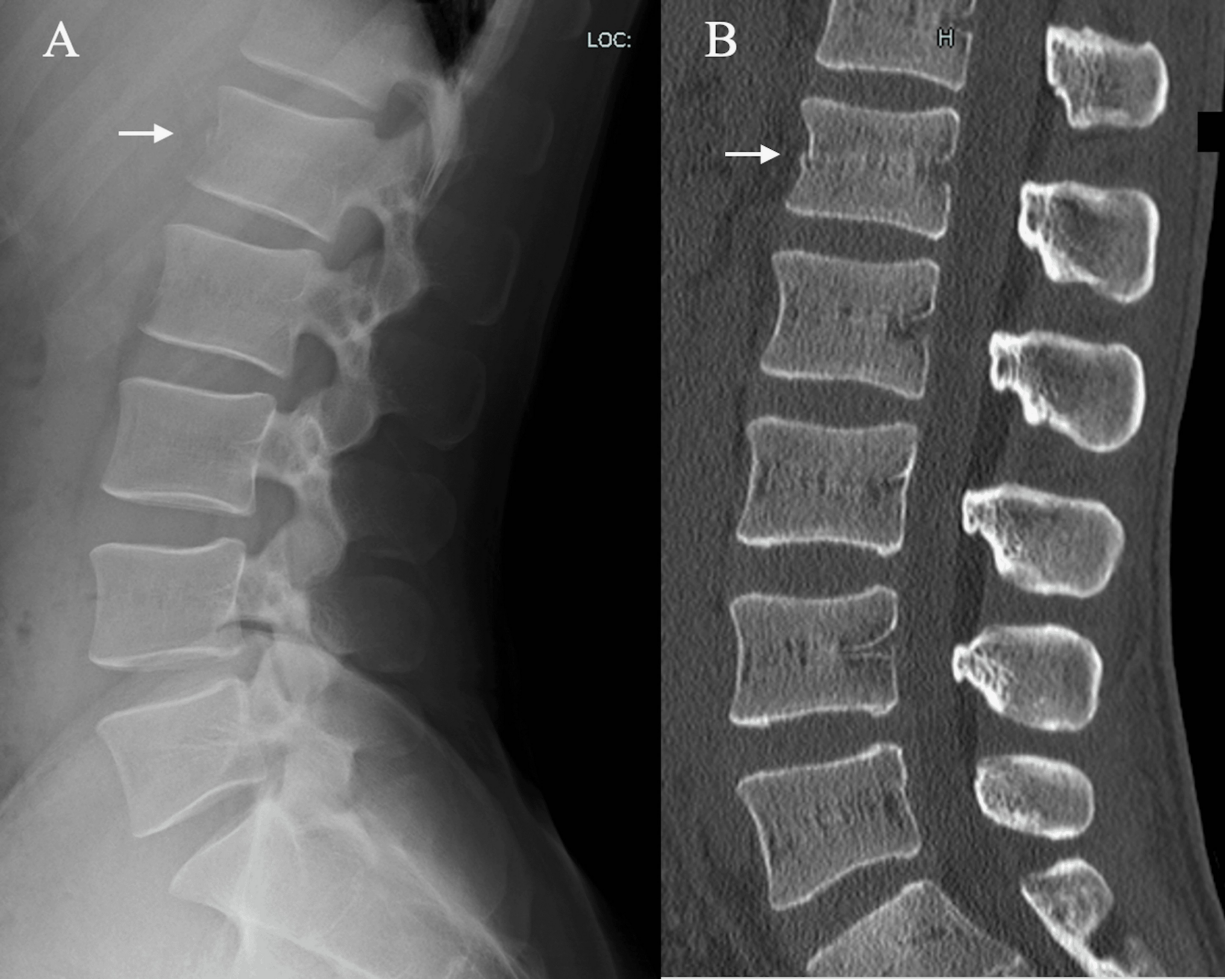
Findings at the day after injury (A) Lateral radiograph showing a fracture of the L1 vertebra. (B) CT sagittal view of the spine showing a fracture of the L1 vertebra (white arrows). CT: computed tomography

A 32-year-old male professional soccer player, in his 14th season as a midfielder, sustained a hyperflexion injury during a mid-air collision, followed by a fall in the middle of a competitive match. He experienced immediate localized lower back pain (LBP) but reported no radiating symptoms, sensory disturbances, or motor deficits. He was immediately removed from play and referred for medical evaluation the following day. Initial radiography and CT revealed a compression fracture of the L1 vertebral body (Figure 1).

**Figure 2:**
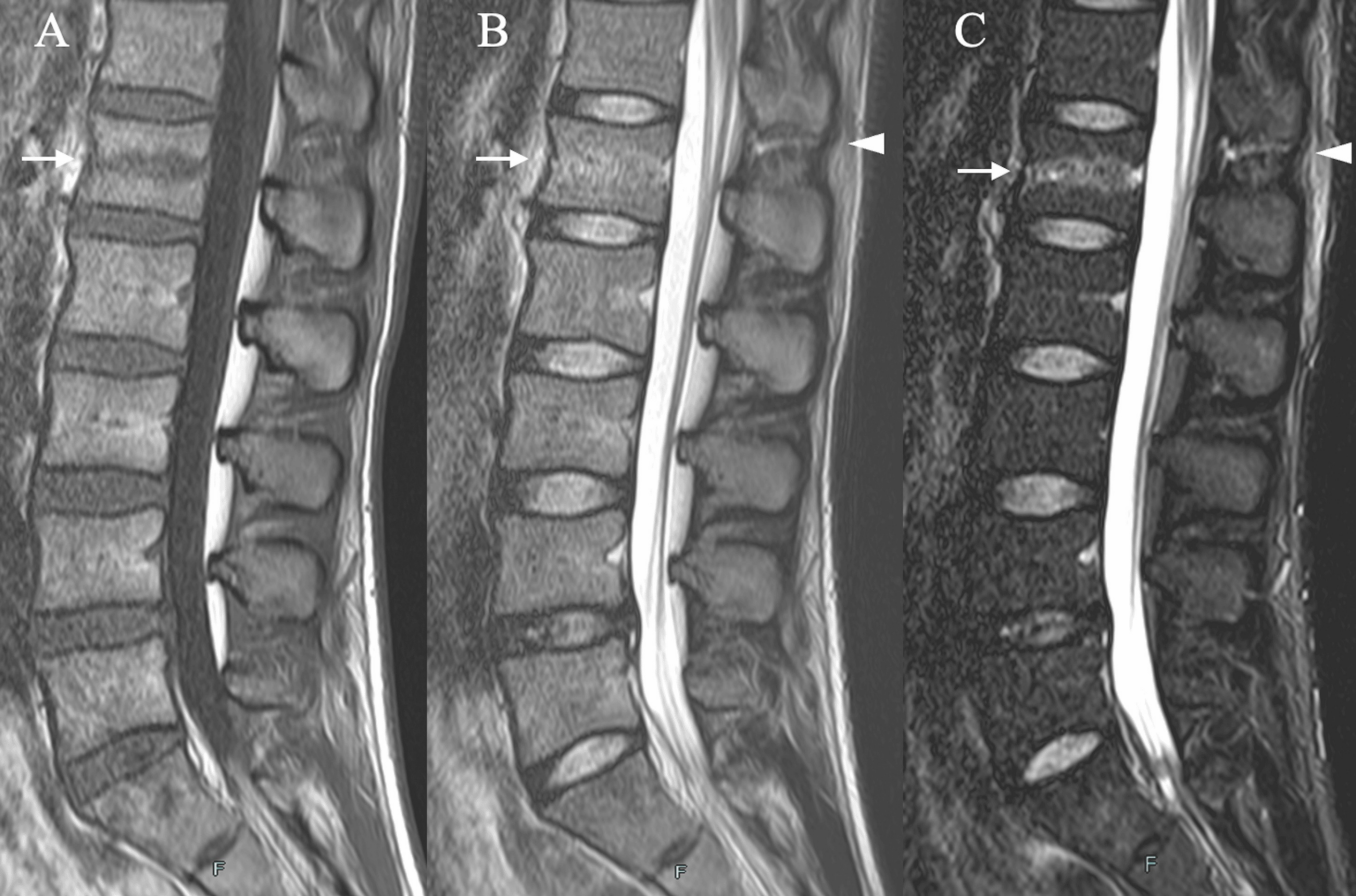
MRI findings (A) T1-weighted (B) T2-weighted (C) STIR MRI sagittal view of the spine showing a fracture of the L1 vertebra (white arrows) and disruption of the supraspinous and interspinous ligaments at the T12–L1 level (arrowhead). MRI: magnetic resonance imaging, STIR: short tau inversion recovery

MRI revealed disruption of the supraspinous and interspinous ligaments at the T12-L1 level (Figure 2), consistent with a flexion-distraction mechanism. The injury was classified as an AO spinal thoracolumbar injury type B2 lesion, which typically requires surgical stabilization.

Despite this, the athletes’ clinical presentation included the absence of neurological symptoms, well-preserved sagittal alignment, minimal intervertebral disc degeneration, and excellent trunk muscle control, as evidenced by a level 5 score on the Sahrmann Core Stability Test [7]. His LBP during movement and the local tenderness were rated 5 on the Numeric Rating Scale. A conservative treatment plan was adopted after multidisciplinary consultation with three sports spine specialists and shared decision-making with the athlete and team management. Surgery would be reconsidered if further vertebral collapse or instability developed.

A rigid thoracolumbar orthosis was prescribed, and the athlete began a structured physical therapy program focused on core stabilization. Thoracolumbar flexion and extension were also restricted. Given the absence of lower-limb tightness, isometric trunk and lower-limb exercises were permitted during the early phase. Three weeks post-injury, he progressed to bodyweight exercises, such as squats, as long as the hip flexion did not compromise the lumbar spine. By week five, the local tenderness had resolved, and trunk motion, specifically lateral flexion and rotation, was pain-free, allowing him to begin light jogging.

**Figure 3:**
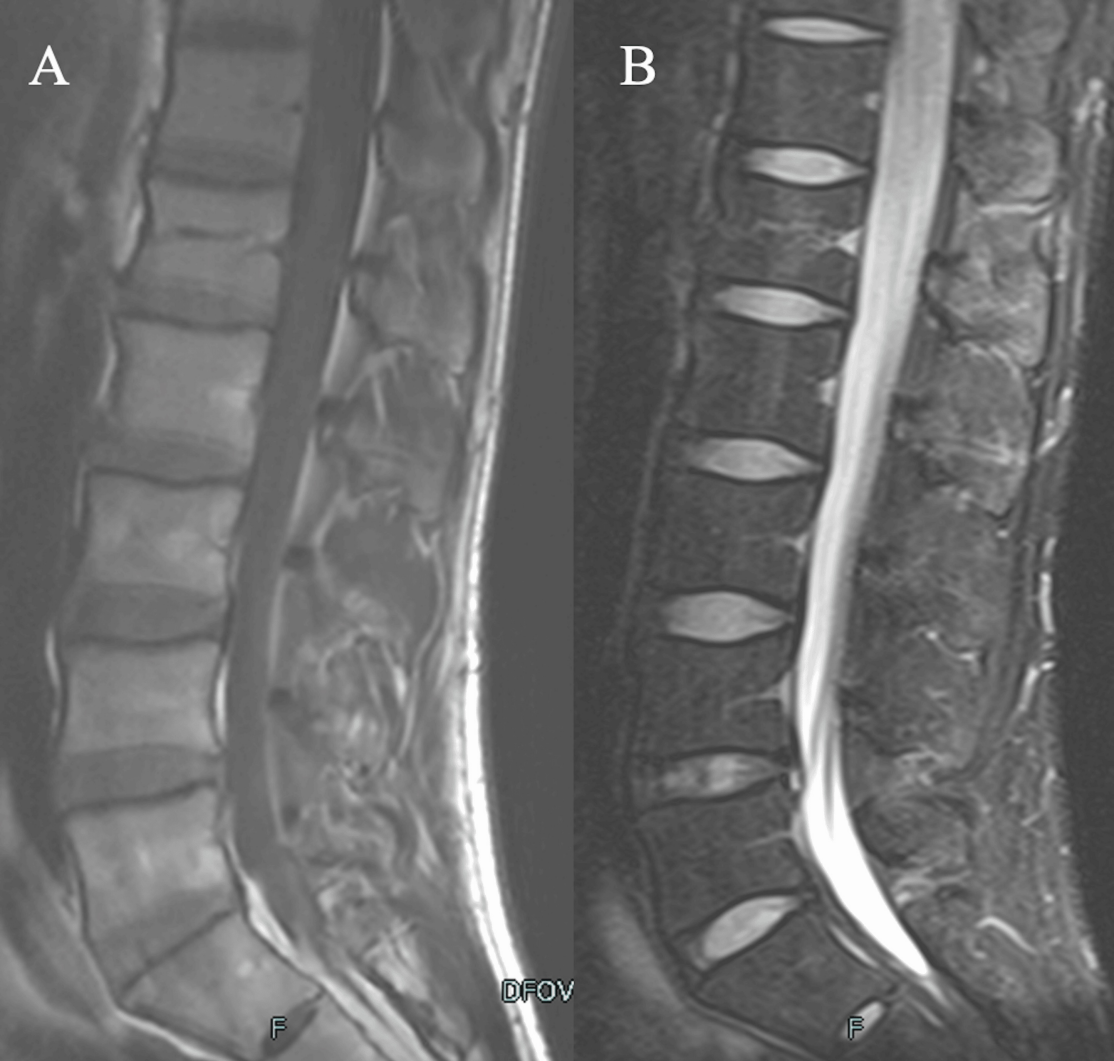
Findings at nine weeks after injury (A) T1-weighted (B) STIR MRI at nine weeks showing resolution of the posterior ligament injury. MRI: magnetic resonance imaging, STIR: short tau inversion recovery

**Figure 4:**
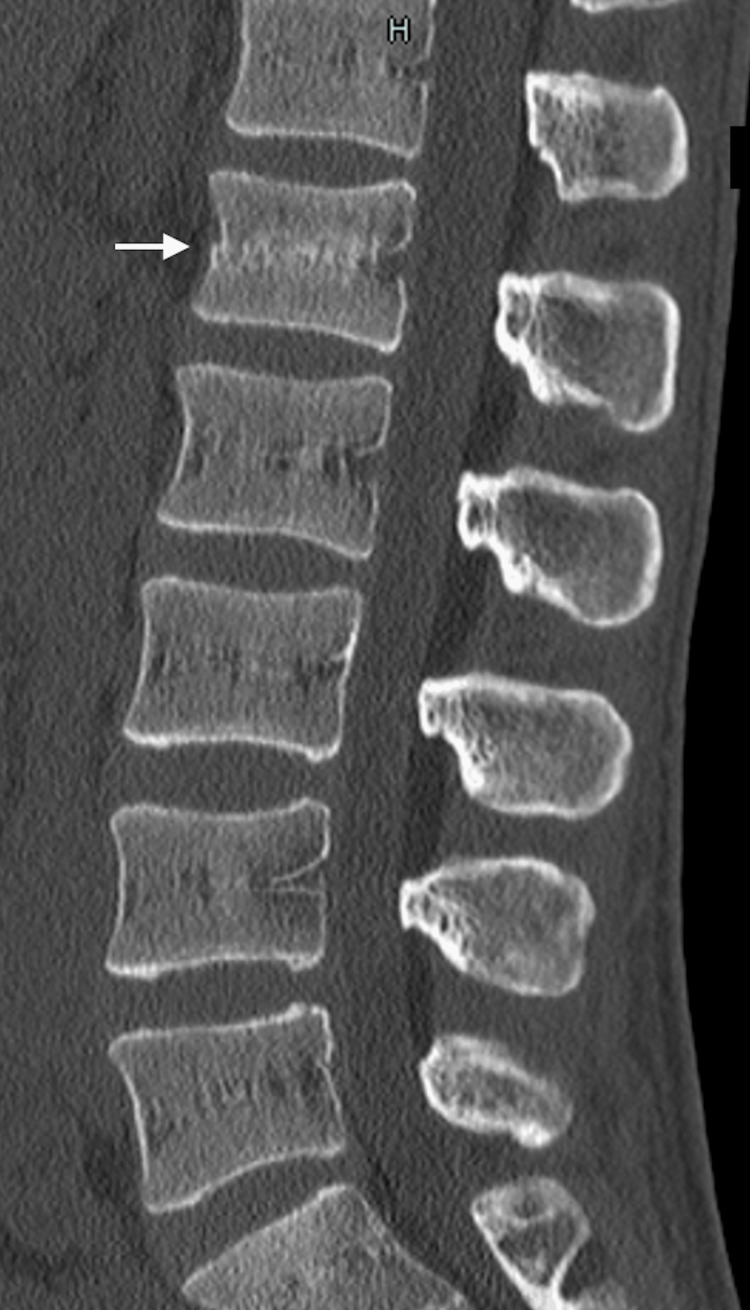
Findings at 10 weeks after injury CT sagittal view of the spine showing vertebral bone union (white arrows). CT: computed tomography

At week seven, the athlete began non-contact ball training. MRI at week nine showed resolution of the posterior ligamentous injury (Figure 3), and CT at week 10 confirmed vertebral bone union (Figure 4).

Orthosis was discontinued, and contact practice was resumed. At 12 weeks, he returned to full match play without restrictions. He completed the season without any re-injury or complications. At the two-year follow-up, he remained asymptomatic with preserved spinal alignment and no evidence of recurrent compression.

## Discussion

This report highlights the rare presentation of a flexion-distraction injury of the lumbar spine in a professional soccer player. Although spinal trauma is relatively rare in athletes, its functional impact can be significant, particularly in sports that require trunk mobility, such as soccer. Flexion-distraction injuries, including Chance fractures, typically result from high-energy mechanisms and are classically associated with motor vehicle collisions involving lap-type seatbelts [8]. However, rare cases have been reported in athletes.

Boham and O'Connell reported a T12 Chance fracture in a rodeo athlete caused by extreme lumbothoracic hyperflexion when the horse bucked within the chute. The athlete underwent surgical stabilization and returned to competition nearly three years later [8]. Similarly, Gotfryd et al. documented a T12 AO type B2 flexion-distraction injury in a professional female soccer player who returned to play six months after posterior fusion surgery [9].

Despite these surgical cases, the literature provides limited guidance regarding RTP criteria after thoracolumbar injuries in elite athletes [10]. Bonsignore et al. reviewed the current evidence and emphasized that RTP decisions must be individualized by incorporating factors such as fracture stability, neurological status, treatment type, and athlete-specific demands [6]. They underscored the lack of standardized timelines and highlighted the need for athletes to be asymptomatic, neurologically intact, and demonstrate full strength and spinal mobility before returning to sports.

Surgical stabilization, while providing mechanical stability, is not without drawbacks. Lumbar fusion, particularly in professional athletes, is associated with reduced spinal flexibility, potential implant complications, and an increased risk of adjacent segment disease [11]. A retrospective study of professional athletes undergoing lumbar fusion demonstrated a mean return-to-play time of 14-24 weeks; however, some athletes failed to return to prior performance levels [10]. Radcliff et al. referred to spinal fusion as potentially "career-ending" for athletes engaged in hypermobile sports [6].

In contrast, our case supports the feasibility of conservative treatment when the following key conditions are met: no neurological deficits, preserved sagittal alignment, minimal disc degeneration, and high-level core control. Through structured rehabilitation, close monitoring, and a staged return to activity, our athletes resumed contact practice by week 10. They returned to full match play by week 12, within or earlier than the surgical RTP range.

This case illustrates that for highly trained athletes with strong musculoskeletal conditioning and favorable injury patterns, nonoperative management can offer a safe and timely return to elite sports, avoiding the long-term drawbacks of spinal fusion. Crucially, successful outcomes depend on careful clinical evaluation, multidisciplinary input, and shared decision-making, underscoring the importance of individualized treatment planning for spinal injuries in professional athletes.

## Conclusions

This case illustrates that conservative treatment may be a safe and effective alternative to surgical fixation in elite athletes with thoracolumbar flexion-distraction injuries. Although the injury was classified as mechanically unstable (AO type B2), the absence of neurological deficits, minimal vertebral collapse, preserved sagittal alignment, and strong core control supported nonoperative management. The athlete returned to full training and competition within 12 weeks of the injury and remained asymptomatic at two-year follow-up. Although fusion remains the standard recommendation for such injuries, its long-term implications, such as loss of mobility and adjacent segment degeneration, are particularly impactful in professional sports. This case highlights the importance of individualized assessment, interdisciplinary consultation, and shared decision-making in the management of spinal trauma in high-level athletes. With appropriate management and monitoring, conservative care may enable an early RTP without compromising spinal integrity or athletic performance.

## References

[1] Zileli M, Sharif S, Fornari M (2021). Incidence and epidemiology of thoracolumbar spine fractures: WFNS Spine Committee recommendations. Neurospine.

[2] Hasler RM, Hüttner HE, Keel MJ, Durrer B, Zimmermann H, Exadaktylos AK, Benneker LM (2012). Spinal and pelvic injuries in airborne sports: a retrospective analysis from a major Swiss trauma centre. Injury.

[3] Bigdon SF, Gewiess J, Hoppe S, Exadaktylos AK, Benneker LM, Fairhurst PG, Albers CE (2019). Spinal injury in alpine winter sports: a review. Scand J Trauma Resusc Emerg Med.

[4] Kane I, Ong A, Radcliff KE, Austin LS, Maltenfort M, Tjoumakaris F (2015). Epidemiology of aquatic and recreational water sport injuries: a case-control analysis. Orthopedics.

[5] Vaccaro AR, Oner C, Kepler CK (2013). AOSpine thoracolumbar spine injury classification system: fracture description, neurological status, and key modifiers. Spine (Phila Pa 1976).

[6] Radcliff KE, Kalantar SB, Reitman CA (2009). Surgical management of spondylolysis and spondylolisthesis in athletes: indications and return to play. Curr Sports Med Rep.

[7] Faries MD, Greenwood M (2007). Core training: stabilizing the confusion. Strength Cond J.

[8] Boham M, O'Connell K (2014). Unusual mechanism of injury resulting in a thoracic chance fracture in a rodeo athlete: a case report. J Athl Train.

[9] Gotfryd AO, Franzin FJ, Hartl R (2016). Thoracolumbar Chance fracture during a professional female soccer game: case report. Einstein (Sao Paulo).

[10] Bonsignore-Opp L, Galivanche A, El Naga AN, Gendelberg D (2024). Return to play criteria after adult lumbar spinal fractures: a review of current literature and expert recommendations. Curr Rev Musculoskelet Med.

[11] Byvaltsev VA, Kalinin AA, Shepelev VV, Pestryakov YY, Aliyev MA, Konovalov NA (2021). Results of minimally invasive lumbar fusion in professional athletes: a single-center retrospective study (Article in Russian). Zh Vopr Neirokhir Im N N Burdenko.

